# Efficacy and safety of laparoscopic holmium laser lithotripsy in the treatment of complicated biliary calculus: A PRISMA-compliant systematic reviews and meta-analysis

**DOI:** 10.1097/MD.0000000000014286

**Published:** 2019-01-25

**Authors:** Penghui Jin, Wutang Jing, Weipeng Zhan, Caiwen Han, Moubo Si, Jia Yang, Yiping Li, Yuanhui Gu, Yuntao Ma, Tiankang Guo

**Affiliations:** a Clinical Medical College, Gansu University of Chinese Medicine, Lanzhou, Gansu; b Gansu Provincial Hospital, Lanzhou, Gansu, China.

**Keywords:** biliary calculus, holmium laser, laparoscopic, lithotripsy, meta analysis

## Abstract

**Backgroud:**

The aim of this study was to assess the efficacy and safety of laparoscopic holmium laser lithotripsy (LHLL) in the treatment of complicated biliary calculus.

**Methods:**

We systematically searched the electronic database (PubMed, EMBASE, Cochrane library, Web of science, and Chinese Biomedical Literature Database) up to May 2018 to identify case-controlled studies that compared LHLL with laparoscopic bile duct exploration (LBDE) for complicated biliary calculus.

**Results:**

Five case-controlled studies were included, with 541 patients (273 in the LHLL group and 268 in the LBDE group). Compared with LBDE, LHLL was associated with shorter operative time (weighted mean difference [WMD] = -40.04, *P* < .001) and lower estimated blood loss (EBL) (WMD = -56.42, *P* < .001), lesser duration of hospitalization (WMD = -3.93, *P* < .001) and lower rate of residual stone (OR = 0.13, *P* < .001). There was no statistically significant differences in bile leakage (OR = 0.48, *P* = .23) and hemobilia (OR = 0.49, 0.41).

**Conclusion:**

Current evidence suggests that the efficacy of LHLL is superior to that of LBDE but they are similarly safe for the treatment of complicated biliary calculus. Limited by the quantity and quality of the studies included, these conclusions need to be verified by more high-quality studies.

## 1 Introduction

Cholelithiasis is a common disease, including gallstone, bile duct stone (common bile duct stone [CBDS] and intrahepatic or extrahepatic bile duct stone), and gallstone with CBDS. The CBDSs are present in about 10–20% of individuals with symptomatic cholecystolithiasis.^[1]^ Many health problems are associated with it, including pain, jaundice, infection, and acute pancreatitis.

Laparoscopic cholecystectomy (LC) has become the 1st choice in the treatment of cholecystolithiasis.^[2]^ However, the treatment methods of bile duct stones are varied, for example, open/minimally invasive surgery or radiological methods.^[1]^ It must be noted, however, that there are many methods to manage cholelithiasis, but so far, no single method has shown significant advantages over any other method.^[3]^ There is also no clear treatment for complicated biliary calculus, which is impacted and large, especially in laparoscopic management.^[4]^ The main difficulty is that increases the risk of biliary bleeding, bile duct injury, or bile duct stenosis, lengthening the surgical incision.

Recent studies have reported that laparoscopic holmium laser lithotripsy (LHLL) has good results in impacted CBDS fragmentation in short series. The study conducted by Varban et al^[5]^ the 1st to investigate the use of holmium laser together with laparoscopic bile duct exploration (LBDE) in the treatment of CBDS, showed that complete stone clearance from the CBD was achieved in all patients without any postoperative complications. A recent study also revealed that stones were completely removed in 8 patients with complex CBDS by 1-stage laparoscopic holmium laser treatment, and that no postoperative complications developed.^[6]^ Similarly, Xia et al also reported that the application of LHLL improved the success rate of LBDE from 63.5% to 93.7%.^[4]^ To sum up, because the optimal laparoscopic management of complex cholelithiasis remains unclear, our study aimed to explore the efficacy and safety of the choledochoscope and LHLL in the treatment of complex cholelithiasis.

## 2 Materials and methods

This study is a systematic review and meta-analysis of previously published studies and does not require ethical approval and patient consent.

This study was conducted and reported based on the Preferred Reporting Items for Systematic Reviews and Meta-analysis.^[7]^

### 2.1 Literature search

We searched the relevant publications in the following electronic databases: PubMed, Embase and Cochrane Library, Web of Science, and the Chinese Biomedical Literature Database from January 1966 to May 2018. The following search/Medical Subject Headings (MeSH) terms were used: “bile duct [MeSH] OR bile vessel OR biliary∗ duct OR Common Bile Duct [MeSH] OR choledoch∗ OR Hepatic Duct [MeSH] OR Common Hepatic Duct” AND “calculi [MeSH] stone OR calculus∗ OR lithiasis OR concretion” AND “holmium laser”. In order to search comprehensively, there were no restrictions on the surgical approach or language used in the study, and references lists were also manually reviewed from selected studies. The last search date was May 25, 2018.

### 2.2 Study selection

#### 2.2.1 Inclusion criteria

The inclusion criteria of this study were based on a “Population, Intervention, Comparison, Outcome, Study” strategy: population, refractory gallstone; Intervention, LHLL; comparison, LBDE; and study, prospective and retrospective observational studies, randomized controlled trial (RCT), case-controlled studies (CCSs), and cohort study (CS).

#### 2.2.2 Exclusion criteria

The exclusion criteria were as follows: reviews, letters, case reports, and conference abstracts; unavailable full text, unavailable data of our interest, literature with the same author, and Newcastle–Ottawa scale score (NOS) < 6.^[8]^

### 2.3 Outcomes of interest

Outcomes of interest in which the efficacy of the 2 techniques was compared were as follows: intraoperative parameters (i.e., operative time, estimated blood loss [EBL], conversion to open procedure/conversion rate, and residual stone rate); postoperative parameters (i.e., length of stay in hospital [LOS], intestinal function recovery time [IFRT], and total hospitalization costs); complications (i.e., bile leakage, hemobilia, stricture of the bile duct, and wound infection).

### 2.4 Data extraction and quality assessment

Two researchers (J.P. and J.W.) who had undergone strict evidence-based medical training independently extracted data, such as the name of the 1st author, year of publication, study design, surgical approach, number of participants, age, sex, stone feature and outcomes of interest. Moreover disagreements were resolved by discussion and consensus. The quality of observational studies was assessed by the modified NOS.^[9]^

### 2.5 Statistical analysis

All statistical analyses were conducted by the Review Manager (RevMan) software, version 5.3. (Copenhagen: The Nordic Cochrane Centre, The Cochrane Collaboration, 2014). Dichotomous variables were expressed as odds ratio (OR), continuous variables were pooled using weighted mean difference (WMD) with 95% confidence intervals (CIs), and a *P*-value less than .05 was considered statistically significant.

Statistical heterogeneity among the meta-analysis was tested using the Chi-squared test.^[10,11]^ In accordance with Higgins’ *I*^2^ statistic, heterogeneities < 25%, 25%∼50%, and > 50% were defined as low, moderate, and high, respectively.^[10]^ A fixed effects model was used to calculate the pooled ORs and WMDs when the heterogeneity was low and moderate; otherwise, a random effects model was used for studies with high heterogeneity.^[12]^ When there was high heterogeneity, we used sensitivity analysis to find its source.

## 3 Result

### 3.1 Study selection

**Figure 1:**
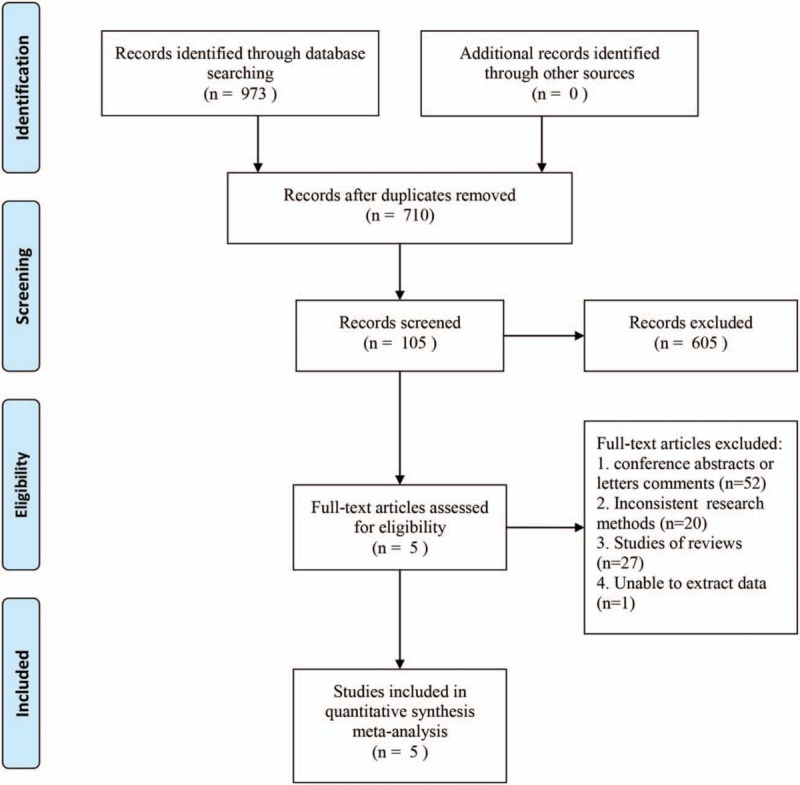
Screening flow chart for the included studies.

A total of 973 related studies were initially obtained in the preliminary literature search. We reviewed 710 results after excluding duplicates by EndNote 6. We finally identified 5 studies that compared LBDE and LHLL in patients with complex biliary calculus, which were gradually and carefully selected by reviewing titles and abstracts and their full text. The literature screening process and results are shown in Figure 1.

### 3.2 Study characterristics

Five CCSs were included, with 541 patients (273 in the LHLL group and 268 in the LBDE group). The basic characteristics of the included studies and risk assessment results of bias are shown in Table 1. The NOS scores of selected studies were as follows: 2 studies with an NOS score of 7 and 3 studies with an NOS score of 6.

**Table 1:**
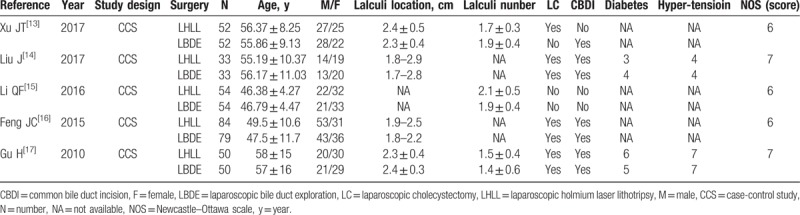
Characteristics of the selected studies included in the meta-analysis.

### 3.3 Intraoperative parameters

#### 3.3.1 Operative time

**Figure 2:**
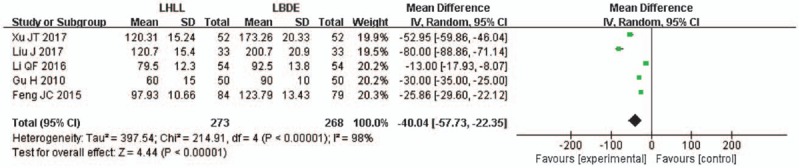
A meta-analysis of operative time for LHLL versus LBDE. LBDE = laparoscopic bile duct exploration, LHLL = laparoscopic holmium laser lithotripsy.

Operative time was described in 5 studies.^[13–17]^ The meta-analysis results of the random effects model showed that the average operative time of 40 min was shorter in the LHLL group and the difference between the 2 groups was statistically significant (WMD = -40.04; 95% CI -57.73, -22.35; *P* < .001), and there was a high heterogeneity among the studies (*I*^2^ = 98%, *P* < .001) (Fig. 2).

#### 3.3.2 EBL

**Figure 3:**
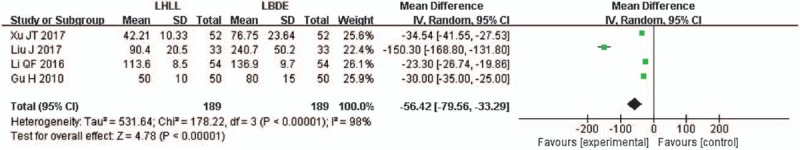
A meta-analysis of estimated blood loss for LHLL versus LBDE. LBDE = laparoscopic bile duct exploration, LHLL = laparoscopic holmium laser lithotripsy.

Four studies reported EBL.^[13–15,17]^ The meta-analysis results of the random effects model demonstrated that EBL was lesser in the LHLL group than LBDE group and the difference between the 2 groups was statistically significant (WMD = -56.42; 95% CI,-79.56, -33.29; *P* < .001), with a high heterogeneity (*I*^2^ = 98%, *P* < .001) (Fig. 3).

#### 3.3.3 Conversion to the open procedure

The conversion rate was reported in only 1 study, with no patient in the LHLL group and 6 patients in LBDE group, and a significant difference was found between the 2 groups.^[17]^

### 3.4 Postoperative parameters

#### 3.4.1 Duration of hospitalization

**Figure 4:**
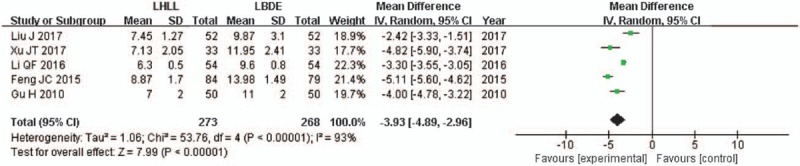
A meta-analysis of duration of hospitalization for LHLL versus LBDE. LBDE = laparoscopic bile duct exploration, LHLL = laparoscopic holmium laser lithotripsy.

Five studies described the duration of hospitalization.^[13–17]^ Pooled data analysis demonstrated that duration of hospitalization was lower in the LHLL group than that in the LBDE group and the difference between the 2 groups was statistically significant (WMD = -3.93; 95% CI, -4.89, -2.96; *P* < .001), and the heterogeneity was high among studies (*I*^2^ = 93%, *P* < .001) (Fig. 4).

#### 3.4.2 Rate of residual stone

**Figure 5:**
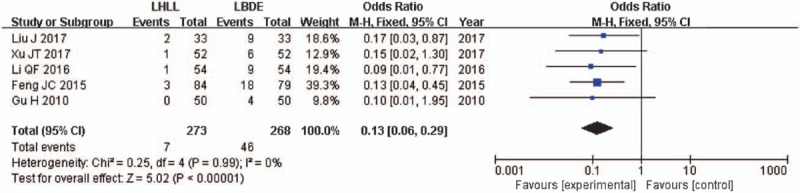
A meta-analysis of rate of residual stone for LHLL versus LBDE. LBDE = laparoscopic bile duct exploration, LHLL = laparoscopic holmium laser lithotripsy.

The residual stone rate was described in 5 studies,^[13–17]^ and the difference between the 2 groups was statistically significant. The meta-analysis results of the fixed effects model further confirmed these (OR = 0.13; 95% CI, 0.06–0.29; *P* < .001) (Fig. 5).

#### 3.4.3 Time to bowel function recovery and total hospitalization costs

Time to bowel function recovery was reported in only one study,^[13]^ which showed that the average recovery time of 1.56 days was lesser in the LHLL group and the difference between the 2 groups was statistically significant (*P* < .05). Moreover, total hospitalization cost was also reported in only 1 study,^[16]^ which revealed that the average cost of 8395.22 CNY was greater in the LHLL group and the difference between the 2 groups was statistically significant (*P* < .05).

### 3.5 Complication

#### 3.5.1 Bile leakage

**Figure 6:**
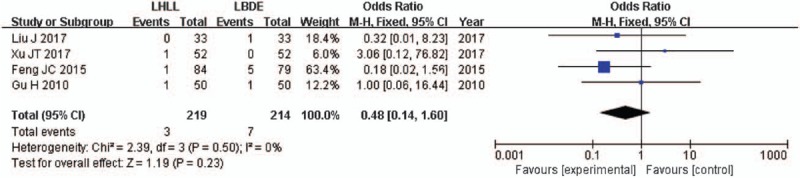
A meta-analysis of bile leakage for LHLL versus LBDE. LBDE = laparoscopic bile duct exploration, LHLL = laparoscopic holmium laser lithotripsy.

Four studies reported on bile leakage.^[13,14,16,17]^ Furthermore, pooled data showed that bile leakage was not significantly different between the 2 groups (OR = 0.48; 95% CI, 0.14–1.60; *P* = .23) (Fig. 6).

#### 3.5.2 Hemobilia

**Figure 7:**
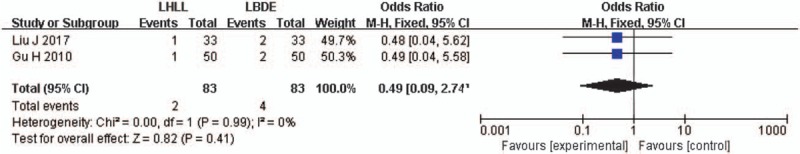
A meta-analysis of hemobilia time for LHLL versus LBDE. LBDE = laparoscopic bile duct exploration, LHLL = laparoscopic holmium laser lithotripsy.

Hemobilia was described in 2 studies.^[14,17]^ Moreover, pooled data demonstrated that bile leakage was not significantly different between the 2 groups (OR = 0.49; 95% CI, 0.09–2.74; *P* = .41) (Fig. 7).

#### 3.5.3 Other complications

Only 1 study reported infection of the biliary tract, wound infection, and liver function injury, and the difference between the 2 groups was statistically significant.^[16]^ Stricture of the bile duct was described in only 1 study and the difference between the 2 groups was not statistically significant.^[17]^

### 3.6 Sensitivity analysis

We performed a sensitivity analysis on high heterogeneity outcomes (i.e., operative time, EBL and duration of hospitalization) by excluding different studies individually. Moreover, the results were not changed, which indicated that these outcomes were robust.

## 4 Discussion

Laser lithotripsy has the advantages of high success and low complication rate in the treatment of ureteral stones.^[18,19]^ Currently, there are different lasers used for stone fragmentation: holmium: YAG, thulium: YAG lasers, KTP: YAG, and LBO, YAG and diode lasers.^[20]^ However, LHLL has greater flexibility, which increases the access to previously unreachable areas, and its visible diode can help target stones and reduce collateral damage.^[21,22]^ The good outcomes obtained with the use of lasers in urinary stones^[23]^ prompted their adoption in the treatment of complex cholelithiasis. This study only explored LHLL in the treatment of complex gallstones.

The results of the meta-analysis showed that the difference in operative time, EBL, duration of hospitalization, residual stone rate between the 2 operation methods were statistically significant. Compared with those in the LBDE group, the operation time was shorter, EBL was lesser and residual stone rate was lower in the LHLL group. However, there was no statistical difference in bile leakage and hemobilia. In addition, infection of the biliary tract, wound infection, liver function injury, and stricture of the bile duct were reported in only 1 study, and quantitative synthetic analysis was not performed.

Operative time, EBL, and duration of hospitalization are important outcome indicators of surgery. This study showed that the mean operative time in the LHLL group was 40 min, which was significantly shorter than that in the LBDE group. Ni et al^[24]^ explored the safety and efficacy of the electronic choledochoscope combined with LHLL in the treatment of complicated cholelithiasis. The results showed that the mean operative time was 67.8 ± 24.8 min, which was similar to the results of this study. Additionally, there were also some findings that are inconsistent with our study. Jun et al^[25]^ showed that the mean operative time was 123 ± 18 min, ranging from 72 to 155 min. The median operative time was 225 min reported by Petersson et al^[4]^. Inconsistent results may be due to the difference in the skill of the surgeons or different LHLL approaches, through the gallbladder duct or choledochal incision.

The mean EBL in the LHLL group was 56 mL, which was significantly lesser than that in LBDE group. A meta-analysis^[26]^ showed that the average EBL was 26.2 mL in the LHLL group. Although the results were inconsistent with those in our study, the difference was not of clinical significance. The mean duration of hospitalization in the LHLL group was 4 days, and similar results were also validated in this study (mean duration of hospitalization was 5 days).

Residual stone rate, bile leakage, and hemobilia are the most important outcome indicators in laparoscopic treatment of cholelithiasis. A meta-analysis showed that, compared with LBDE, LHLL could effectively reduce the residual stone rate. In addition, the meta-analysis results also revealed that LHLL could reduce the incidence of major complications (e.g., bile leakage and hemobilia), although there was no statistical difference. Similar results can also be obtained from some case analyses.^[24,25,27]^ And some studies have shown that in patients in whom clearance of CBDS has been unsuccessful (despite the use of techniques including mechanical lithotripsy and ERCP with prior sphincterotomy), cholangioscopy-guided holmium laser lithotripsy using endoscopic procedures results in very high stone clearance rates (73–97%).^[28–30]^ Therefore, the safety of the two surgical methods is similar.

Limitations of this study: 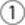 The quality of the methodology of the included study was generally low, mainly because there was no RCT for the comparison of the 2 procedures and only retrospective case-control studies were available, and all of the studies were from China, which inevitably led to selective, implementation, and measurement biases. 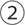 Most of the studies did not adequately report complications, which might lead to selective outcome reporting bias. 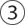 There was significant heterogeneity in some outcome indicators and the causes might be the difference in the type of holmium laser instrument, size and quantity of stone, etc. 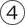 The difference in the surgeon's operative technique, proficiency in the use of machines, and auxiliary staff level will inevitably influence the result. However, the present study data are not sufficient to conduct a subgroup analysis, and more research is needed to provide data support.

## 5 Conclusion

Current evidence suggests that the efficacy of LHLL is superior to that of LBDE but they are similarly safe for the treatment of complicated biliary calculus. Limited by the quantity and quality of the studies included, these conclusions need to be verified by more high-quality studies.

## Author contributions

Conception and design of the study: Tiankang Guo and Yiping Li. Studies selection: Penghui Jin, Wutang Jing and Jia Yang. Data extraction: Caiwen Han, Yuntao Ma and Moubo Si. Statistical analyses: Penghui Jin and Wutang Jing. Wrote the paper: Penghui Jin and Yuanhui Gu. The paper was revised and approved by Penghui Jin.

**Conceptualization:** Weipeng Zhan.

**Data curation:** Weipeng Zhan, Jia Yang, Yuntao Ma.

**Formal analysis:** Penghui Jin, Wutang Jing, Weipeng Zhan.

**Investigation:** Caiwen Han, Moubo Si, Yuanhui Gu.

**Methodology:** Penghui Jin, Wutang Jing.

**Project administration:** Jia Yang, Yuntao Ma.

**Resources:** Caiwen Han, Moubo Si, Yuanhui Gu.

**Software:** Penghui Jin, Wutang Jing.

**Supervision:** Yiping Li, tiankang guo.

**Validation:** Yiping Li, tiankang guo.

**Writing – original draft:** Penghui Jin.

**Writing – review & editing:** Yiping Li, tiankang guo.
